# Identification of interacting transcription factors regulating tissue gene expression in human

**DOI:** 10.1186/1471-2164-11-49

**Published:** 2010-01-19

**Authors:** Zihua Hu, Steven M Gallo

**Affiliations:** 1 Center for Computational Research, New York State Center of Excellence in Bioinformatics & Life Sciences, Department of Biostatistics, Department of Medicine, State University of New York (SUNY), Buffalo, NY 14260, USA; 2 Center for Computational Research, New York State Center of Excellence in Bioinformatics & Life Sciences, State University of New York (SUNY), Buffalo, NY 14260, USA

## Abstract

**Background:**

Tissue gene expression is generally regulated by multiple transcription factors (TFs). A major first step toward understanding how tissues achieve their specificity is to identify, at the genome scale, interacting TFs regulating gene expression in different tissues. Despite previous discoveries, the mechanisms that control tissue gene expression are not fully understood.

**Results:**

We have integrated a function conservation approach, which is based on evolutionary conservation of biological function, and genes with highest expression level in human tissues to predict TF pairs controlling tissue gene expression. To this end, we have identified 2549 TF pairs associated with a certain tissue. To find interacting TFs controlling tissue gene expression in a broad spatial and temporal manner, we looked for TF pairs common to the same type of tissues and identified 379 such TF pairs, based on which TF-TF interaction networks were further built. We also found that tissue-specific TFs may play an important role in recruiting non-tissue-specific TFs to the TF-TF interaction network, offering the potential for coordinating and controlling tissue gene expression across a variety of conditions.

**Conclusion:**

The findings from this study indicate that tissue gene expression is regulated by large sets of interacting TFs either on the same promoter of a gene or through TF-TF interaction networks.

## Background

Transcriptional regulation in eukaryotic organisms is a fundamental process to determine a gene's spatial and temporal expression. One of the main events involved in this process is the binding of TFs to short DNA motifs, called transcription factor binding sites (TFBSs), on the promoter regions of genes, activating or repressing the transcription machinery. In mammalian tissues most TFs do not act alone, but work through combinatorial regulation [1,2], in which two or more TFs work synergistically to control individual gene expression. This combinatorial regulation is able to increase the specificity and flexibility of genes in controlling tissue development and differentiation. Therefore, one of the major first steps toward understanding how tissues achieve their specificity is to identify interacting TFs regulating gene expression in different tissues.

Early attempts to identify interacting TFs controlling tissue gene expression came from the use of experimental approaches such as gel retardation assay [3], site-directed mutagenesis [4], chromatin immunoprecipitation [5,6], and genomic microarrays [5,6] in tissues such as liver [3,5-8], pancreas [6], immune systems [9,10], muscle [11-13], and neural stem cells [14]. In these studies, interactions between TFs were discovered on a limited scale. To overcome this limitation, some researchers built models to predict tissue-specific *cis*-regulatory modules in liver [15,16] and muscle [17] tissues. Taking advantage of the unprecedented amount of sequence and gene expression information from the most recent technical and experimental advances, a few researchers have developed computational approaches to predict tissue-specific TFs and *cis*-regulatory modules based on recognizable sequence features from either highly expressed genes [18] or genes expressed only in a particular tissue [19-21] derived from genome-wide gene-expression profiling. Some of these researchers have defined tissue-specific enhancers by combining gene-expression profiling, genome comparison, and TFBS analyses [18] or have predicted TF synergy using the relative position and co-occurrence of TFBSs in the promoters of genes expressed only in a particular tissue [19]. Others have looked for tissue-specific *cis*-regulatory modules by enrichment analysis for motifs discovered *de novo *in tissue-specific promoters relative to other promoters from the same species [21]. Despite all these efforts, the mechanism that determines tissue development and differentiation is still not fully understood, as the regulation of tissue gene expression involves complex combinatorial interactions between TFs.

In this study, rather than using sequence features of promoters from genes that are expressed only in a particular tissue [19-21], we used our function conservation approach [22] to predict interacting TFs from the most highly expressed genes in each of 79 human tissues [23]. Our approach predicts interacting TFs by integrating the function conservation of interacting TFs from both their binding sites and target genes between closely related species, which are based on the following two assumptions. The first is based on the strong possibility that functional TFBS pairs have more distance constraint than random co-occurrence of TFBSs. The second relies on the biological assumption that while a TF pair plays the same role in regulating gene expression between closely related species, the occurrence of its binding sites is expected to be more highly enriched in promoter sequences of orthologous genes than in promoter sequences of non-orthologous genes. Other than function conservation, the use of highly expressed genes in a tissue allows one to avoid the elimination of common genes contributing to tissue development and differentiation between tissues, especially for closely related tissues (e.g. skeletal muscle and heart), when compared to the use of tissue-specific genes [19-21] which are expressed only in a particular tissue. To our knowledge, this is the first use of a function conservation approach and highly expressed genes in tissues for interacting TF prediction. Therefore, the findings provide novel insight into how tissue gene expression is controlled.

The application of the function conservation approach to the most highly expressed genes has led to the prediction of hundreds of interacting TFs from each of the 79 human tissues. Based on these predictions, TF pairs associated with a certain tissue were identified. The validity of these discovered TF pairs has been evaluated by both known interacting and liver-specific TFs. We further extended our study to find interacting TFs controlling gene expression in a broad spatial and temporal manner by looking for TF pairs common to the same type of tissues, from which TF-TF interaction networks were further built. As a first step to elucidating *cis*-regulatory modules involved in tissue gene regulation, we also performed analysis to identify interactions of 3 TFs.

## Results

### Overall analysis procedures

**Figure 1:**
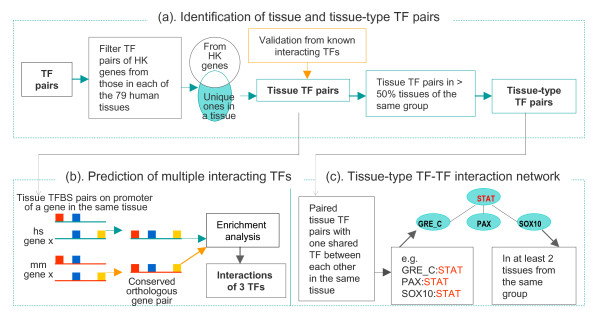
**Flowchart of analysis procedures**. (a) Identification of tissue and tissue-type TF pairs. (b) Prediction of interactions of three TFs. (c) Reconstruction of tissue-type TF-TF interaction networks. HK: housekeeping genes; hs: human; mm: mouse.

The overall analysis procedures are shown in Additional File 1 and Figure 1. We employed our previously developed function conservation approach [22] to first search for TF pairs using the top 300 expressed genes (referred to tissue-expressed genes) [18] in each of the 79 human tissues from the GNF Atlas2 gene expression database (gnfAtlas2) [23] and their corresponding mouse orthologous genes (Additional File 1). We also utilized promoter sequences from 1018 human housekeeping genes [24] and their mouse orthologous genes to predict interacting TFs playing ubiquitous roles in different tissues (see Methods).

We next filtered out the TF pairs in a particular tissue common to those from housekeeping genes (Figure 1a). The remaining TF pairs (referred to tissue TF pairs) in each tissue were more tissue-specific. The rationale for this filtering is that in each tissue some of the interacting TFs play general roles, since all tissues possess common mechanisms to control the fundamental biological processes. To find interacting TFs controlling tissue gene expression in a broad spatial and temporal manner, we extended the analysis to identify tissue TF pairs common to the same type of tissues (referred to tissue-type TF pairs) as well as interactions of 3 TFs. For the former, we looked for common tissue TF pairs in at least 50% tissues of the same type (Figure 1a). We also built TF-TF interaction networks by joining 2 or more tissue-type TF pairs with one shared TF between TF pairs in the same tissue. TF-TF interaction networks with the same topology in at least 2 tissues from the same tissue type were defined as "tissue-type TF-TF interaction networks" (Figure 1c). Finally, a two-step analysis of TFBS conservation and enrichment of overlapping TF target orthologous genes was performed to predict interactions of 3 TFs (Figure 1b).

### Identification of tissue TF pairs

Using the function conservation approach [22] and tissue-expressed genes in each of the 79 human tissues, we were able to identify a few hundred TF pairs for each tissue, for which BM-CD71+early erythroid has the largest number of 383 TF pairs, and the ovary tissue has the smallest number of at 230. We also identified 647 TF pairs from housekeeping genes. Filtering TF pairs of housekeeping genes from those in each tissue has greatly reduced the number of TF pairs in each tissue, ranging from 39% TF pairs for lymph node to 59% TF pairs for BM-CD105+endothelial, indicating that a large portion of the TF pairs performs ubiquitous roles across different tissues. The resulting tissue TF pairs range from 111 to 176 for different tissues. We also searched for TF pairs specific to one tissue (referred to tissue-unique TF pairs) and obtained from 2 to 20 such TF pairs for different tissues. The number of tissue TF pairs and tissue-unique TF pairs for each of the 79 human tissues are summarized in Table 1. The top 5 tissue TF pairs that have the most significant correlations between enriched TFBS pairs and enriched overlapping orthologous genes are also listed for each tissue.

**Table 1:** Summary of the identified tissue and tissue-unique TF pairs as well as top 5 tissue TF pairs in the 79 human tissues.

Tissue	# tissue TF pair	# tissue Unique TF pair	Top 5 tissue TF pairs	Literature support
Fetal liver	150	17	HNF3:HNF4ALPHA**, MYOGNF1:PPARA*, PPARA:PAX2*, HNF1:OCT4*, CMAF:COUP_DR1*	[4]
**Liver**	162	18	CEBPGAMMA:HNF4ALPHA**, HNF4ALPHA:HNF4ALPHA**, CEBPGAMMA:CEBPGAMMA*, AIRE:HNF3*, HNF3B:RUSH1A*	[3,26,41]
**Fetal lung**	149	9	CEBP:HNF4ALPHA**, CEBP:CEBPA**, VDR:OCT, CEBPGAMMA:PLZF, FOXJ2:GATA4	[3,41]
**Lung**	111	8	EBOX:HNF4ALPHA**, CEBP:ETS**, SP3:WT1*, CACD:CETS1P54, EBOX:SPZ1	[42,43]
**Kidney**	173	15	HNF3:HNF4ALPHA**, PPARA:SP3, AP2:HAND1E47, AP2:ER, TEL2:SREBP	[4]
**Pancreas**	146	14	HNF3:HNF4ALPHA**, AP2:TBP*, DEC:MYOGNF1, GATA4:PAX4*, HNF4ALPHA:YY1	[4]
**Pancreatic islets**	125	9	SP1:SREBP1**, E2A:ZF5_B*, CP2:CP2, CHOP:OCT1, OCT:RUSH1A	
**Cardiac myocytes**	139	12	HNF3:HNF3B**, CEBP:PAX4, POU3F2:PAX4, NFAT:SP3, TST1:SREBP1	[4]
**Heart**	130	10	MYOGNF1:SP3*, CP2:ZIC3, AP2:TAXCREB, TAL1BETAE47:MAF, FAC1:OCT1	
**Skeletal muscle**	121	15	OCT1:SP1**, MYOGNF1:DR4*, NKX25:TBP*, TEL2:ZIC3, AP2ALPHA:CP2	[44]
**Smooth muscle**	137	10	HMGIY:OCT**, EGR1:MYOGNF1*, TST1:PAX2, AP2:TST1, POU3F2:CEBP	
**Tongue**	152	17	CEBP:CEBPA**, HNF4ALPHA:SREBP1, MYOGNF1:VDR*, HIC1:MYOGNF1*, CP2:ZIC3	[41]
**Uterus**	144	12	AP1:HMGIY**, HAND1E47:ZIC3*, PLZF:YY1*, AIRE:PLZF, SP3:WT	[45]
**Uterus corpus**	158	17	HMGIY:OCT**, HNF3:MYOGNF1*, CP2:E2A*, NF1:SP1*, OCT4:TGIF	[46]
**Ciliary ganglion**	149	15	AP1:STAT**, CACD:CETS1P54, MYOGNF1:NFY, CETS1P54:VDR, AP2:OCT4*	[47,48]
**Dorsal root ganglion**	155	15	CEBP:CEBPGAMMA**, FOXJ2:DR3, AREB6:FOXJ2, CEBP:TST1*, AP2ALPHA:GEN_INI3_B*	[41]
**Spinal cord**	144	6	ETS:HMGIY**, HIC1:PPARA*, NKX25:PLZF, PPARA:TBP*, CART1:MYOGNF1	[10]
**Superior cervical ganglion**	157	15	CEBPGAMMA:HNF4ALPHA**, AP1:PLZF*, ETF:HOXA4*, FAC1:GATA4, DR3:TBP*	[3]
**Trigeminal ganglion**	166	18	ETS:VDR**, TEL2:SPZ1, MINI19_B:PLZF, AP2ALPHA:PAX4*, CP2:TST1*	[49]
**Amygdala**	148	7	OCT1:OCT1**, PAX:STAT*, EGR1:PAX2, AP2:CETS1P54*, MRF2:HMGIY	[50,51]
**Caudate nucleus**	161	6	CEBPGAMMA:CEBPGAMMA**, ETS:VDR**, AP2ALPHA:KROX*, GATA4:XVENT1, CEBPGAMMA:HMGIY	[41]
**Cerebellum**	149	11	CEBPGAMMA:HMGIY, VDR:TAXCREB*, OSF2:PAX2, DR4:SPZ1*, FAC1:OCT4*	
**Cerebellum peduncles**	123	6	CEBPGAMMA:CEBPGAMMA**, ETS:MYB**, ETS:VDR*, CART1:FAC1, DR4:SPZ1*	[41]
**Cingulate cortex**	131	3	EBOX:ETS**, ETS:HMGIY**, NKX25:TBP*, MRF2:OCT4* AP2:XVENT1*	[10,52,53]
**Fetal brain**	121	12	ETS:HMGIY**, MINI19_B:SRY*, AHRARNT:VDR*, CACD:TAXCREB*, CDXA:HMGIY	[53]
**Globus pallidus**	145	8	ETS:VDR**, NKX25:TBP*, NKX25:PAX5, AHRHIF:KROX*, AP2:SREBP*	[49]
**Hypotalamus**	134	5	ETS:HMGIY**, CEBPGAMMA:CEBPGAMMA**, AP2:PPARA*, AP2:PPARA*, PAX:SREBP1*	[41,53]
**Medulla oblongata**	124	3	VDR:TAXCREB*, CEBPGAMMA:HMGIY, AP2:ETF*, MAF:PAX4*, MYOGNF1:ZIC3	
**Occipital lobe**	132	6	AP2:XVENT1*, NKX25:TBP*, AP2:PPARA*, AP2ALPHA:KROX*, PAX:STAT*	
**Olfactory bulb**	155	7	CEBPA:GRE_C, AP2:TST1*, OCT:TST1*, CEBPGAMMA:HMGIY, AP2:PAX4*	
**Parietal lobe**	125	2	EBOX:ETS**, TTF1:VDR, AP2:XVENT1*, NKX25:TBP*, CP2:HIC1	[52]
**Pons**	160	3	ETS:HMGIY**, OCT1:OCT1**, ETS:VDR**, GATA:GATA4*, AP2:SREBP*	[50,53]
**Prefrontal cortex**	131	6	PAX:STAT*, AP2:XVENT1*, CMAF:SP3, TAL1BETAE47:PAX2, AHRHIF:KROX*	
**Subthalamic nucleus**	128	5	OCT:PAX5*, NKX25:TBP*, AP2ALPHA:DR4*, AHRHIF:KROX*, CP2:ZIC3	
**Temporal lobe**	138	8	AP2:ETF*, PAX3:SP1*, POU3F2:MYOGNF1*, NKX25:TBP*, CETS1P54:HMGIY	
**Thalamus**	137	11	PAX:STAT*, CEBPGAMMA:GEN_INI3_B, XVENT1:YY1, MAZ:VMYB, AP2ALPHA:PLZF*	
**Whole brain**	130	8	CEBPGAMMA:CEBPGAMMA**, GATA:OCT4*, AP2:PPARA*, AP2:PPARA*, POU3F2:NFAT*	[41]
**BM CD105+ endothelial**	143	10	OCT:STAT**, CEBPGAMMA:PLZF*, ER:TBP*, GRE_C:PPARA, M YOGNF1:SP1*	[54]
**BM CD34+**	158	7	ETS:HMGIY**, CEBPGAMMA:PLZF*, ETF:TST1*, ETS:RUSH1A*, TAL1BETAE47:SP3*	[53]
**BM CD71+ earlyerythroid**	145	8	PAX4:YY1*, GEN_INI3_B:GEN_INI3_B, KROX:NF1*, NF1:SP1*, CP2:CP2	
**BM_CD33+ myeloid**	164	16	CEBP:CEBPA**, ETS:VDR**, AP2ALPHA:EGR1* P300:SREBP1, CETS1P54:VDR*	[41,49]
**Bone marrow**	154	16	CEBP:CEBPA**, DR4:SPZ1*, AP2:OCT4, VDR:SREBP*, DR3:WT1*	[41]
**Lymph node**	157	7	MINI19_B:LRF, OCT4:PAX4, CP2:ZIC3, AP2ALPHA:TTF1, TAL1BETAE47:PPARA*	
**PB BDCA4+ dentritic cells**	159	9	CEBPA:CEBPGAMMA**, KROX:PPARA*, CEBP:TST1*, TST1:PAX2*, DR3:P300*	[41]
**PB CD14+ monocytes**	146	9	CEBP:CEBPA**, TFE:TST1*, NF1:PAX8, NF1:ZIC3, MYOGNF1:SP1*	[41]
**PB CD19+ Bcells**	143	5	HMGIY:OCT**, DBP:TBP*, ETS:HOXA4*, CP2:CP2, RUSH1A:RUSH1A	[46]
**PB CD4+ Tcells**	161	10	ETS:SP1**, CEBPGAMMA:CEBPGAMMA**, CP2:SZF11, FAC1:VMYB, AREB6:GATA4	[41,55]
**PB CD56+ NKCells**	125	10	ETS:GRE_C, CEBPA:CEBPGAMMA**, OCT1:SPZ1* CEBP:TST1*, AP2:GRE_C	[41]
**PB CD8+ Tcells**	155	8	ETS:SP1**, AREB6:GATA4, AP2ALPHA:MAZ, TTF1:OCT1_07*, KROX:XVENT1*	[55]
**Thymus**	164	11	EBOX:HNF4ALPHA**, CEBPA:CEBPGAMMA**, AP1:STAT**, ETS:RUSH1A*, TBP:YY1*	[41,42]
**Tonsil**	158	10	CEBP:CEBPGAMMA**, NF1:P300, CACD:TAL1BETAE47*, PPARA:SP1*, OSF2:CDXA	[41]
**Whole blood**	132	8	CEBPGAMMA:CEBPGAMMA**, PPARA:SP3*, ETS:VMYB*, SP3:YY1*, NFAT:PLZF*	[41]
**Ovary**	127	9	CEBPA:SREBP1, GATA_C:MAF, LRF:NKX25, NF1:ZIC3, CHOP:PAX4	
**Testis**	149	9	CEBPGAMMA:CEBPGAMMA**, DR4:SPZ1*, POU3F2:PAX4, MYOGNF1:ZIC3, MYOGNF1:SP1	[41]
**Testis germ cell**	136	8	HAND1E47:SPZ1*, SP3:WT1, DBP:TBP, TEL2:LRF, POU3F2:COUP_DR1	
**Testis interstitial**	115	5	CEBP:CEBPGAMMA**, MYOGNF1:SPZ1*, DR4:SPZ1*, CEBPGAMMA:HMGIY, EBOX:SREBP1	[41]
**Testis leydig cell**	135	6	MYOGNF1:SPZ1*, FAC1:FOXJ2, TTF1:PPARA, GATA4:RUSH1A, HOXA4:OCT	
**Testis seminiferous tubule**	157	9	STAT:STAT**, DR4:SPZ1*, FAC1:FOXJ2, AHRHIF:AP2ALPHA, CMAF:PPARA	[56]
**Adrenal cortex**	147	17	CEBP:NFAT**, PPARA:SP1, HMGIY:ZF5_B, AP2:TST1, AP2ALPHA:TST1	[57]
**Adrenal gland**	157	8	PAX3:WT1, PPARA:SP3, TAL1BETAE47:CRX, DR3:SP3, CETS1P54:OCT	
**Fetal thyroid**	127	9	CEBPA:CEBPGAMMA**, TST1:PAX2, CART1:PPARA, DR3:SP3, AP2:PPARA	[41]
**Pituitary gland**	133	19	EBOX:P300, OSF2:YY1, AP2:PPARA, SP3:WT1, EGR1:ZF5_B*	
**Prostate**	176	12	CEBPA:ETS**, EBOX:ETS**, ETS:VDR**, AP1:DR3, KROX:NF1	[43,58]
**Salivary gland**	132	11	CEBPGAMMA:CEBPGAMMA**, AP2:POU3F2, TTF1:TTF1, CEBPA:CEBPGAMMA, EGR1:P300	[41]
**Thyroid**	122	11	CEBPA:CEBPGAMMA**, PPARA:SREBP1, GRE_C:SREBP1, MINI19_B:DR3, CMAF:YY1	[41]
**721 B lymphoblasts**	168	11	CART1:YY1, CP2:ZIC3, HMGIY:PAX4, DBP:TTF1, TEL2:P300	
**Colorectal adenocarcinoma**	143	11	FOXJ2:EFC, CART1:HOXA4, MINI19_B:DR4, SP3:WT1, OCT4:PAX2	
**Leukemia chronic myelegenous**	151	9	TCF11:NFAT, NCX:PAX2, ETF:SRY, NF1:ZIC3, AP2ALPHA:AREB6	
**Leukemia lymphoblastic**	156	17	AP2ALPHA:PAX4, CEBPA:PLZF, AP2ALPHA:TST1, AP2:PPARA, HMGIY:ZF5_B	
**Leukemia promyelocytic**	154	6	CEBPGAMMA:CEBPGAMMA**, DR4:SPZ1, CEBPA:FAC1, P300:ZIC3, CP2:ZIC3	[41]
**Lymphoma burkitts daudi**	147	12	ETS:MYB**, CETS1P54:WT1, CP2:P300, CP2:EBOX, MINI19_B:WT1	[59]
**Lymphoma burkitts Raji**	131	5	NFAT:OCT1**, PPARA:SP3, E2A:MYOGNF1, CETS1P54:MYB, AREB6:CDPCR3	[60,61]
**Adipocyte**	142	14	PPARA:SP1, EGR1:ZF5_B, SP3:SP3, AP2:SRY, PPARA:WT1	
**Appendix**	136	8	CEBP:NFAT**, ETS:VDR**, TAL1BETAE47:TEL2, SP3:WT1, DR4:SP3	[57]
**Atrioventricular node**	140	13	ETS:GRE_C, AREB6:PPARA, ETF:HOXA4, GEN_INI3_B:GEN_INI3_B, AP2:OCT4	
**Bronchial epithelial cells**	152	14	TTF1:SP1, POU3F2:GATA4, AP2ALPHA:TTF1, CACD:MAZ, CEBPA:GATA4	
**Placenta**	132	5	CEBPA:CEBPGAMMA**, SP3:WT1*, DEC:PAX5, NKX25:STAT1, PPARA:SP1	[41]
**Skin**	156	20	CEBPA:CEBPGAMMA**, HNF4ALPHA:PPARA, HMGIY:OCT4, CEBPGAMMA:PLZF, AP2ALPHA:CP2	[41]
**Trachea**	146	12	CEBPA:CEBPGAMMA**, CEBP:PAX4, CETS1P54:PPARA, AP2:XVENT1, AP2ALPHA:TST1	[41]

Top 5 tissue TF pairs are those with the most significant correlation between enriched TFBS pairs and enriched overlapping orthologous genes;

**experimentally proven interacting TFs with literature support; * at least one TF is tissue-specific based on tissue-specific single TFs from TRANSFAC11.4 database [37]

Overall, we identified 2549 tissue and 803 tissue-unique TF pairs for the 79 human tissues. These results indicate that tissue gene expression is regulated by large sets of interacting TFs. Furthermore, the relative small number of tissue-unique TF pairs out of all tissue TF pairs suggests that identical tissue TF pairs in different tissues may play different functional roles, which prompted us to investigate their biological function. For this purpose, we used Gene2go http://www.ncbi.nlm.nih.gov/ to annotate human genes whose promoters contained the target TFBS pairs, as TFs control cellular biological processes via transcriptional regulation of groups of genes with similar functions. Significant (*q*-value < 0.1) biological processes for tissue TF pairs were obtained by comparing the number of TF target genes involved in a particular biological process to the number of genes for the same biological process in the whole human genome (Fisher's exact test; *p *= 2.4 × 10^-4 ^to 8 × 10^-28^). All tissue and tissue-unique TF pairs as well as their potential biological functions are listed in Additional File 2.

### Evaluation by known interacting TFs

**Figure 2:**
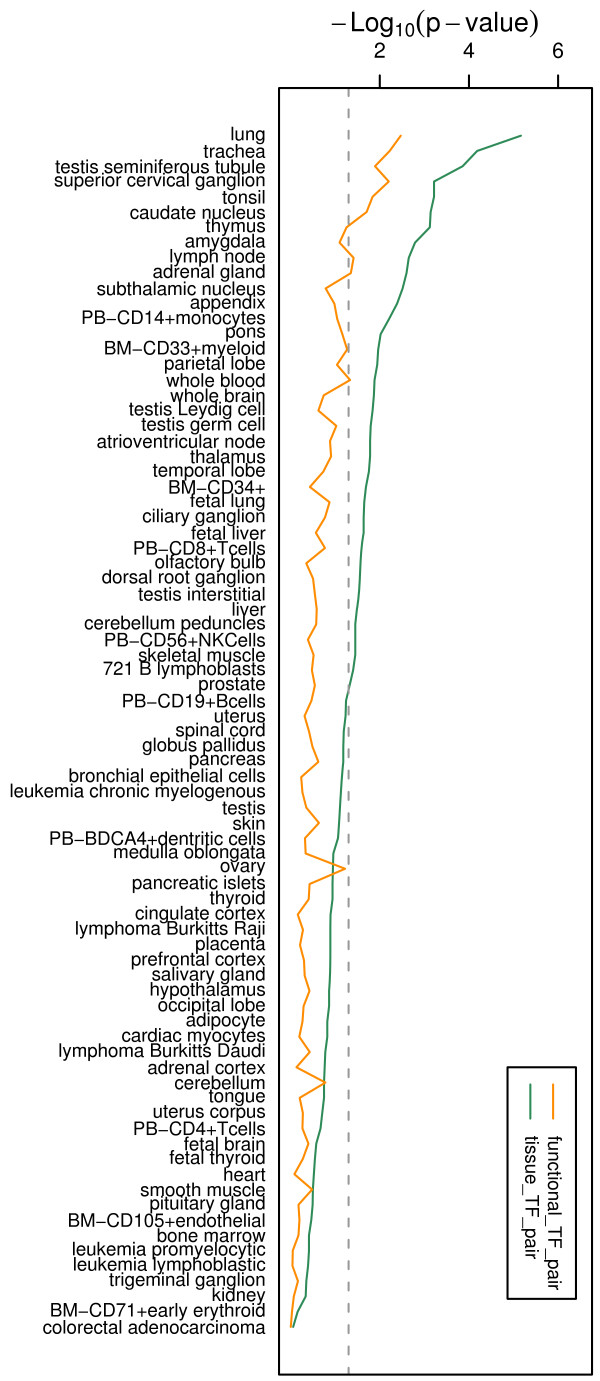
**Statistical significance for the occurrence of known interacting TFs**. Validation results (-log10(*p*-values)) for predicted TF pairs (orange) and tissue TF pairs (green) in the 79 human tissues. Dash line indicates significant *p*-value cutoff which is < 0.05 above the dash line. Known interacting TFs display enrichment more in the tissue TF pairs than in predicted TF pairs for both the number of tissues (37 vs. 9) and the degree of enrichment (Binomial test: *p *= 3.2 × 10^-2 ^to 6.8 × 10^-6 ^vs. *p *= 4 × 10^-2 ^to 3.4 × 10^-4^).

Although the function conservation approach has been proven to be a successful means for predicting interacting TFs [22], we sought to assess the validity of the identified tissue TF pairs. We first used TRANSCompel^® ^10.4 [25] to determine if known interacting TFs were statistically enriched in the predicted tissue TF pairs. The TRANSCompel^® ^database contains 180 experimentally proven composite elements of two or more binding sites which were previously identified by individual wet lab studies from others. Of the180 composite elements, 105 were mapped to the 23,005 (214*215/2) possible combinations of 2 TFs from the 214 non-redundant position weight matrices (PWMs). We first investigated the statistical significance for the occurrence of known interacting TFs in both predicted TF pairs (before filtering) and tissue TF pairs in each of the 79 tissues. Figure 2 shows that known interacting TFs display enrichment more in the tissue TF pairs then in predicted TF pairs for both the number of tissues (37 vs. 9) and the degree of enrichment (Binomial test: *p *= 3.2 × 10^-2 ^to 6.8 × 10^-6 ^vs. *p *= 4 × 10^-2 ^to 3.4 × 10^-4^). We also computed the occurrence of known interacting TFs in all predicted TFs pairs (before filtering) and all tissue TF pairs from the 79 tissues. We found that 40 (38.1%) of the 105 known interacting TFs were in both predicted TF pairs (Binomial test; *p *= 6.4 × 10^-9^) and tissue TF pairs (Binomial test; *p *= 5.4 × 10^-11^).

**Figure 3:**
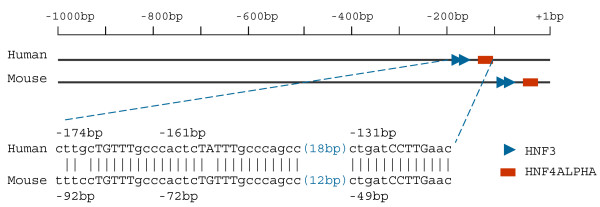
**Conservation of identified HNF3 and HNF4ALPHA binding sites in human and mouse APOA1 genes**. Both schematic and sequence alignments for the predicted HNF3 and HNF4ALPHA binding sites between human and mouse promoter sequences are depicted. In the sequence alignment the core motifs are shown in upper case letters and the distances between adjacent binding sites are shown in brackets. Also shown are the locations of each binding site in relation to the transcriptional starting site.

To further verify our prediction, we next compared the tissue TF pairs to known tissue-specific TFs from liver, for which the *cis*-regulatory systems for both individual TF binding and synergistic actions have been thoroughly studied [3,4,25,26]. These studies found 40 liver-specific single TFs and 27 liver-specific interacting TFs. We first computed the tissue TF pairs whose two TFs were all liver-specific based on the 820 (40*41/2) possible combinations of 2 TFs from the 40 liver-specific single TFs. Out of 162 tissue TF pairs from liver tissue, we were able to obtain 30 (Binomial test; *p *= 2.3 × 10^-14^) where both TFs were liver-specific. For the 27 liver-specific known interacting TFs, we found 8 (30%) in both the predicted TF pairs (Binomial test; *p *= 3.6 × 10^-9^) and tissue TF pairs (Binomial test; *p *= 2.9 × 10^-11^) from liver tissue. These include HNF4ALPHA:HNF4ALPHA, NF1:COUP_DR1, CEBPGAMMA:HNF4ALPHA, CEBPA:HNF3B, HNF3:HNF4ALPHA, HNF3:PPARA, CEPBA:GATA4, and HNF1:OCT1. All of them are key elements in liver specific transcriptional regulation. GO enrichment analyses indicated that genes whose promoters contained the predicted liver-specific TFBS pairs were mainly involved in liver specific functions [27,28], including oxidation reduction, acute-phase response, gluconegnesis, and lipoprotein & lipid metabolic processes (Additional File 2). Further analysis of the binding sites on the promoter sequence of individual genes indicated that we were able to reliably identify interacting TFs similar to those previously reported. One of the examples was the APOA1 gene, which was well-characterized to be synergistically bound by HNF3 and HNF4 [8]. Our prediction was able to successfully identify the HNF3:HNF4 binding sites on its promoter. A closer examination shows that our predicted HNF3 and HNF4 binding sites for the APOA1 gene are exactly those experimentally proven, liver tissue-specific HNF3 and HNF4 binding site combinations [8], which are highly conserved between human and mouse in regards to both nucleotide sequence and spacing between each binding site (Figure 3).

It is important to note that the 79 human tissues represent only part of the temporal and spatial conditions from which the 105 known interacting TFs were discovered, and therefore it is unlikely to have all known interacting TFs in our predicted list. Nevertheless, our results indicate that the use of function conservation approach and tissue-expressed genes was able to reliably identify to a great extent known interacting TFs, thus presenting very strong evidence for the validity of the identified tissue TF pairs. These results also indicate that filtering the TF pairs of housekeeping genes from those in each tissue is an important step to eliminate TFs playing a ubiquitous role, thereby the resulting TF pairs are more tissue-specific.

### Identification of tissue-type TF pairs

**Figure 4:**
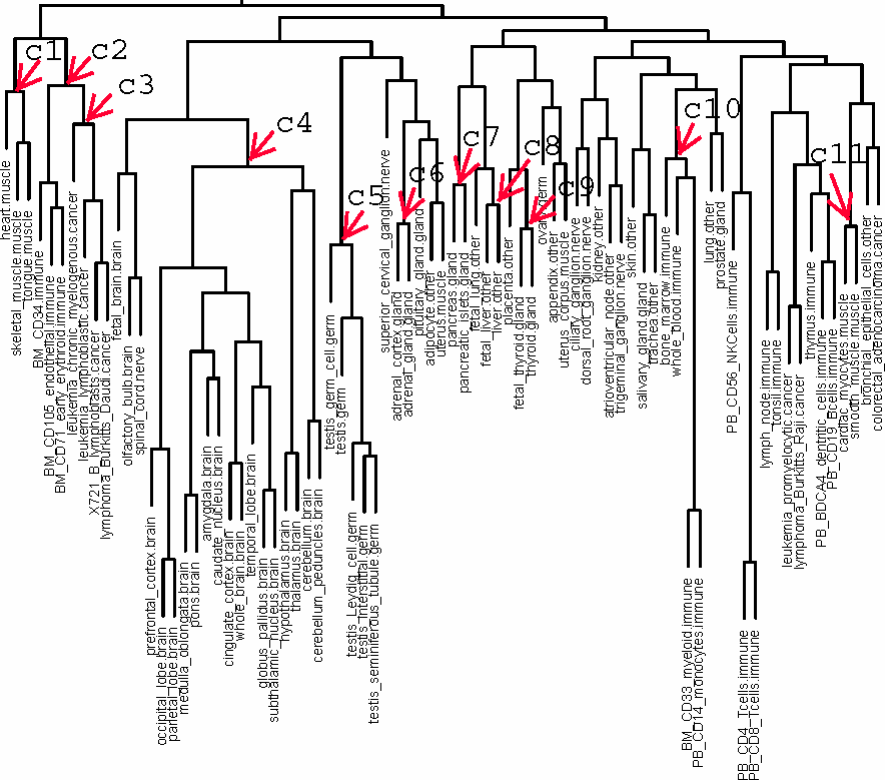
**Hierarchical clustering over tissue TF pairs from the 79 human tissues**. The distance matrix was built using the "binary" method, and hierarchical clustering was performed using the "complete" agglomeration method. Arrows and numbers indicate the selected tissue groups for further analysis.

One of the goals of this study is to find interacting TFs controlling gene expression in a broad spatial and temporal manner such as interacting TFs common to the same type of tissues. This can be achieved by searching tissue TF pairs common across all tissues of the same type such as the 7 muscle tissues. However, the use of all tissues may reduce the power for tissue-type TF pair identification, since the contents of tissue TF pairs and even the function of a common tissue TF pair may be different between tissues of the same type. Therefore, we sought to first classify tissues into smaller but more closely related groups based on tissue TF pairs, from which representative tissues for the same tissue type could be obtained. Accordingly, we used hierarchical clustering to group tissues, as no *a priori *knowledge was available for the number of groups for each tissue type. The results are shown in Figure 4, where tissues of the same type are generally grouped together such as testis, liver, pancreas, and brain. There are, however, exceptions for other tissue types which are grouped into either distinct groups or into groups with other types of tissues such as muscle and immune systems. While the muscle tissues are classified into two distinct groups, of which one contains skeletal muscle, heart, and tongue and the other contains smooth muscle and cardiac myocytes, tissues for immune systems are classified into a few groups, one of which displays tighten link with a few cancer tissues.

**Figure 5:**
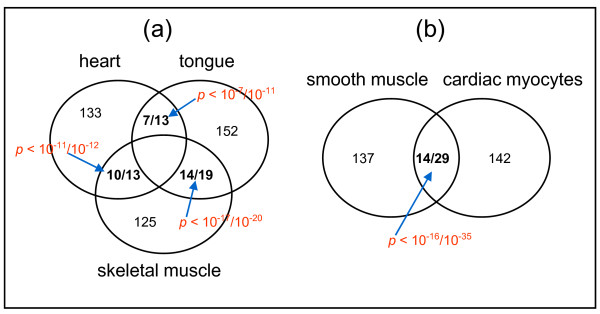
**Overlapping tissue TF pairs and their functions between muscle tissues**. (a) Venn diagram displaying the significant overlap for both tissue TF pairs and their functions in the group of skeletal muscle. (b) Significant overlap for both tissue TF pairs and their functions in the group of smooth muscle. Each circle indicates the number of tissue TF pairs. The number of overlapping tissue TF pairs and TF functions between two tissues is indicated in bold (# function/# TF). Also shown are their corresponding *p*-values from hypergeometric tests.

We extended our analysis to investigate conservation for tissue TF pairs between tissues of the two muscle groups. We computed overlap for both tissue TF pairs and their biological functions between tissues using hypergeometric distribution. We found little or no overlap for both tissue TF pairs and their functions among tissues between these two groups, which was especially true for the function of tissue TF pairs (data not shown). On the other hand, both tissue TF pairs and their functions showed significant overlap between tissues within the same group (Figure 5). These results not only demonstrate the validity of our tissue classification but also indicated that tissues from the same type (here 5 muscle tissues) may have great difference in both the contents of tissue TF pairs and TF functional roles.

Based on the clustering results, we selected 11 tissue-type groups, each having 2 to 16 tissues, for tissue-type TF pair discovery. A cutoff threshold of tissue TF pairs common in at least 50% tissues from the same group was set up for searching tissue-type TF pairs. In addition to the TF level, we also searched for tissue-type TF pairs based on their function using the same criteria of > 50% tissues in the same group. To this end, we were able to identify tissue-type pairs for all tissue groups as listed in Table 2. Whereas the number of tissue-type TF pairs ranges from 17 for immune/cancer group to 74 for testis, those at the functional level have relatively smaller numbers, ranging from 3 for thyroid to 40 for testis. All (379) tissue-type TF pairs as well as their corresponding functions for the 11 tissue-type groups are listed in Additional File 3.

**Table 2:** Number of tissue-type TF pairs in the selected 11 tissue groups.

Tissue type	# Tissues	Cluster ID	# TF pairs	# TF pairs with annotated function
Adrenal gland*	2	C6	31	16
Brain	16	C4	45	26
Cancer	4	C3	32	25
Immune/cancer	3/4	C2	17	14
Immune	4	C10	33	15
Liver	2	C8	30	22
Pancreas	2	C7	23	6
Smooth muscle*	2	C11	29	14
Skeletal muscle*	3	C1	39	26
Testis	5	C5	74	40
Thyroid	2	C9	27	3

* One tissue from the same group is used as the tissue type name.

Cluster ID: clustering ID for the 11 selected tissue groups in Figure 4.

### Reconstruction of tissue-type TF-TF interaction networks

**Figure 6:**
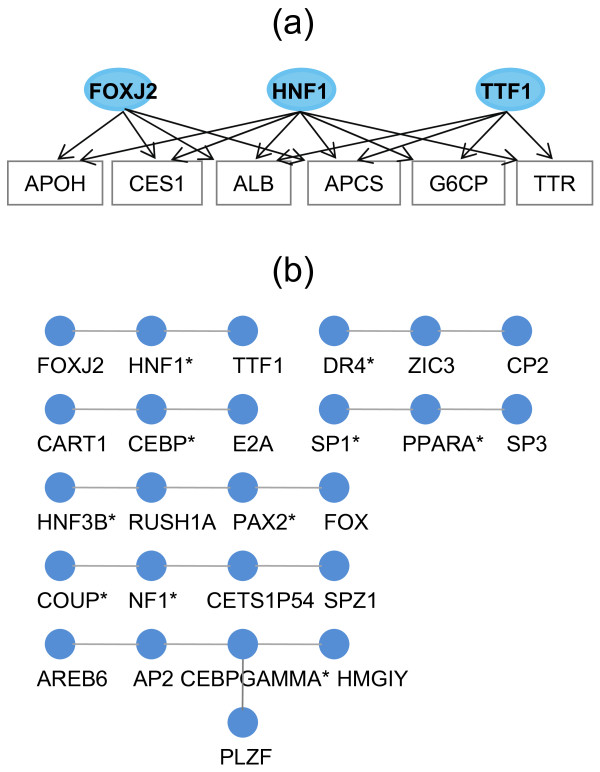
**Tissue-type TF-TF interaction networks for liver tissues**. (a) Multi-input network motif for TFs of FOXJ2, HNF1, and TTF1 and their target genes which are regulated by either two or three TFs. (b) Topology of seven tissue-type TF-TF interaction networks for liver tissues. Each node represents a TF and each edge (link) represents a significant synergy between two TFs from tissue TF pairs. Previously known liver-specific TFs are labeled with an asterisk.

In an effort to reveal TF relationships in controlling tissue gene expression, we performed analysis to reconstruct TF-TF interaction networks. Using tissue-type TF pairs, we first looked for those with one shared TF between each other in the same tissue, from which TF-TF interaction networks were built by joining 2 or more TF pairs (Figure 1c). TF-TF interaction networks with the same topology in at least 2 tissues from the same tissue-type group were then selected as tissue-type TF-TF interaction networks, which are multi-input network motifs consisting of at least 3 TFs that bind to a set of gene promoters. A total of 84 tissue-type TF-TF interaction networks were identified for the 11 tissue-type groups, ranging from 1 for immune/cancer to 22 for testis (Additional File 4). Sixty two of these tissue-type TF-TF interaction networks have a linear relationship between TFs with 1 to 4 internal TFs (i.e. TF connecting to 2 other TFs), indicating that the majority of the TF-TF regulatory networks have simple TF relationships for controlling tissue gene expression. Figure 6a shows a multi-input network motif from liver tissues, in which FOXJ2, HNF1, and TTF1 regulate 6 genes in a combinatorial manner by either 2 or 3 TFs. The remaining 22 tissue-type TF-TF interaction networks display more complex interacting structures with some of the internal TFs connecting to 3 or more TFs.

Unlike the tissue TF pairs, we did not find any common tissue-type TF-TF interaction networks between different tissue types. In light of this, we performed a search to see if any single TFs played central roles in controlling tissue gene expression across different tissues, and looked for internal TFs in multiple tissue-type TF-TF interaction networks. To this end, we found that AP2, PPARA, PAX4, FAC1, ZIC3, and SPZ1 served as internal TFs in 8, 8, 8, 6, 5, and 4 tissue-type TF-TF interaction networks, respectively, suggesting their role as central hubs in tissue-type TF-TF interaction networks. Whereas FAC1 acts as the internal TF in 6 tissue-type TF-TF interaction networks from immune systems and cancer, SPZ1 mainly serves as the internal TF in tissue-type TF-TF interaction networks from testis, and the rest in 5 to 6 tissue-type TF-TF interaction networks from different tissue types. These results indicate that FAC1, when serving as the internal TF, is restricted to the two related tissue types, and that SPZ1, a bHLH-Zip protein, has an important role in testis [29,30]. The rest have more diversified roles for coordinating network TFs in controlling tissue gene expression.

It is interesting to note that no single TFs serve as the central hub for tissue-type TF-TF interaction networks from liver tissue. However, we observed that 6 of 7 tissue-type TF-TF interaction networks had at least one known liver-specific TF serving as the internal TF as shown in Figure 6b. To investigate if this distribution pattern of liver-specific TFs in the TF-TF interaction networks had any biological meaning, we randomly sampled TFs from the 214 PWMs to build TF-TF networks, each having the same size and order as the real TF-TF interaction networks. The simulated TF-TF networks were then compared to tissue-type TF-TF interaction networks to estimate the statistical significance for the distribution of liver-specific TFs. The results indicate that known liver-specific TFs were significantly enriched as internal TFs for these 7 tissue-type TF-TF interaction networks (bootstrap analysis; *p *< 10^-20^). By contrast, the total number of liver-specific TFs in these 7 tissue-type TF-TF interaction networks was not enriched (bootstrap analysis; *p *= 0.11). These results suggest that liver-specific TFs, other than initiating liver-specific transcriptional event, may play an important role in recruiting non-liver-specific TFs to the tissue-type TF-TF interaction network, thus offering the potential for coordinating and controlling gene expression across a variety of conditions.

### Prediction of multiple interacting TFs

As a first step to elucidate *cis*-regulatory modules involved in tissue gene regulation, we extended our analysis to the interactions of 3 TFs (named as multiple interacting TFs). Using tissue TF pairs from each of the 79 tissues, we performed a two-step analysis of TFBS conservation and enrichment of overlapping orthologous genes between human and mouse (see Methods). Although it is likely that multiple interacting TFs may be under estimation by the use of tissue TF pairs instead of all predicted TF pairs, the predicted multiple interacting TFs are tissue-specific. Therefore, these predictions most likely represent *cis*-regulatory modules involved in tissue gene regulation. To this end, we identified 1735 unique interactions of 3 TFs for the 79 human tissues, ranging from 9 multiple interacting TFs for testis interstitial to 72 multiple interacting TFs for caudate nucleus (Additional File 5).

The validity of these predicted multiple interacting TFs was assessed by using liver-specific single TFs from TRANSCFAC11.4 [25], as few known *cis*-regulatory modules were available. We performed analysis to see if known liver-specific TFs were statistically enriched in 30 predicted multiple interacting TFs from liver tissue. We found 4 of them (bootstrap analysis; *p *< 10^-3^) whose 3 TFs were all liver-specific, 18 (bootstrap analysis; *p *< 10^-8^) with at least 2 liver-specific TFs, and 28 (bootstrap analysis; *p *< 10^-5^) with at least 1 liver-specific TF. These results provide evidence for the enrichment of liver-specific TFs in the predicted multiple interacting TFs, which in turn demonstrated the validity of the prediction.

We next searched for all predicted multiple interacting TFs and their potential functions that are common between tissues. The results indicated that, although common multiple interacting TFs existed between most tissues, the highest overlap was within brain tissues and between brain and gland tissues. By contrast, there was little overlap for the functions of multiple interacting TFs, except within brain and cancer and between these 2 tissue types (Additional File 6). The latter is especially interesting to us, as cancer cells have a global effect on immune systems, which in turn control and shape developing cancer [31]. Six multiple interacting TFs were found to have common functions between immune systems and cancer tissues, including CEBPGAMMA:NKX25:PLZF, CEBPGAMMA:PAX4:PLZF, CP2:NFY:PAX4, FOXJ2:PAX4:POU3F2, CEBPGAMMA:PAX4:PLZF, and FOXJ2:HNF3:PAX4. These results revealed not only the common mechanisms for transcriptional regulation but also the common functional role of multiple interacting TFs between cancer and immune systems, including cell cycle, cell division, DNA replication, mitosis, phosphoinositide-mediated signaling, and immune response. These findings therefore provide new insight into the molecular interplay between cancer and immune systems.

## Discussion

Tissue gene expression is generally regulated by multiple transcription factors. A major first step toward understanding how tissues achieve their specificity is to identify interacting TFs regulating gene expression in different tissues. Previous computational approaches to predict interacting TFs were mainly based on recognizable sequence features of tissue-specific [19-21] genes derived from genome-wide gene-expression profiling. Despite these studies, the mechanisms controlling tissue gene expression are still not fully understood.

In this study, we utilized our previously developed function conservation approach, which, based on this and a prior study [22], was shown to successfully predict interacting TFs from tissue-expressed genes. Based on the predictions, tissue TF pairs were identified. The advantage of our approach lies in the fact that it does not depend solely on sequence features of genes but rather function conservation of interacting TFs from both their binding sites and putative target genes between closely related species. Other than function conservation, the use of tissue-expressed genes would allow one to avoid the elimination of common genes contributing to tissue development and differentiation between tissues, especially for these closely related tissues (e.g. skeletal muscle and heart) when compared to the use of tissue-specific genes [19-21] which are expressed in a particular tissue. Therefore, the utilization of our function conservation approach and tissue-expressed genes provides an alternative way for tissue interacting TF discovery.

One of the findings of our study indicates that tissue gene expression is controlled by large sets of tissue TF pairs, which is in agreement with previously reported findings from an approach using sequence features of tissue-specific genes by Yu *et al*. [19]. We were curious to know the differences of interacting TFs identified by the two different approaches, and selected the liver tissue for comparison. For the 8 known liver-specific interacting TFs that were successfully predicted by our approach in the 162 liver tissue TF pairs, we found that HNF3:HNF4ALPHA was in the liver-specific TF pairs predicted by Yu *et al*. However, we did not find the other 7 known liver-specific interacting TFs predicted in our 162 tissue TF pairs from Yu *et al*. On the other hand, 6 of the 27 known liver-specific interacting TFs were correctly predicted by Yu *et al *but were not in our tissue TF pairs from liver tissue. A closer examination shows that liver-tissue TF pairs from our prediction are enriched with CEBP, HNF3, and HNF4, and that liver-specific TF pairs from Yu *et al *are enriched with HNF1 and HNF4. All these TFs are known liver-specific TFs such as HNF3 [32], which initiates the liver transcriptional event, and HNF1 [33], which interacts with other important TFs to establish transcriptional hierarchy in liver tissues. These results demonstrate that different methods were able to identify interacting TFs from different angles. Therefore, the findings from our study provide new insight into the mechanism controlling tissue gene expression.

Filtering TF pairs of housekeeping genes from those of tissue-expressed genes is an important step to eliminate TF pairs which play general but not tissue-specific roles in individual tissues. The filtering process reduced the number of predicted TF pairs from 3024 to 2549 (15.7%) for all 79 tissues. This reduction for TF pairs was, however, significantly larger when individual tissues were concerned (39% to 59%), indicating that a large number of overlapping TF pairs had ubiquitous roles among different tissues. The remaining interacting TFs in each tissue were more tissue-specific, which was best evidenced by the result that the predicted TF pairs from liver tissue contained the same number of known liver-specific interacting TFs before and after the filtering. The relative small number of tissue-unique TF pairs out of all tissue TF pairs and the findings from conservation analysis for the functions of tissue TF pairs between tissues of two muscle groups from this study also indicate that tissue TF pair with identical 2-TF combination might play different functional roles in different tissues.

Our findings show that tissue gene expression is controlled by a variety of interacting TFs either on the promoter of a gene or through TF-TF interaction networks. These identified TF interactions may constitute a large part of interacting TFs in each tissue but is not a complete list. To fully understand the mechanisms controlling tissue gene expression requires additional study, which has been best evidenced from the comparison of interacting TFs in liver tissue between Yu *et al*. [19] and ours. Other than the prediction methods, the target gene selection can contribute greatly to tissue TF identification. Our prediction picked up 8 of the 27 known liver-specific interacting TFs in liver tissues. A couple factors might be responsible for not identifying the other known liver-specific interacting TFs. First, these known liver-specific TF interactions were discovered from broad spatial and temporal conditions. The selected liver genes in this study however represented only one of many conditions under which liver-specific TFs play their roles. This was exemplified by known liver-specific interacting TFs in tissue TF pairs from liver and fetal liver tissues from our prediction. Whereas tissue TF pairs from liver tissue contained 8 known live-specific interacting TFs, fetal liver contained 3 known live-specific interacting TFs with 2 common to those in liver, demonstrating the impact of temporal conditions on tissue TF discoveries. Second, it is unlikely for the top 300 tissue-expressed genes from a single condition to all have information for tissue interacting TF prediction. The choice of the top 300 tissue-expressed genes was based on the report of Pennacchio *et al*. [18] who have successfully used them to predict tissue-specific enhancers. Increasing the size of genes however would increase the chance of bringing noise to the prediction. Therefore, other than different computational approaches, selecting a proper list of tissue-expressed genes would have a great impact on the prediction of tissue TF pairs.

One of the goals of this study was to find interacting TFs controlling tissue gene expression in a broad spatial and temporal manner. We performed analysis to identify tissue-type TF pairs for 11 selected tissue-type groups. While, as described above, each specific tissue may reflect only a small portion of all spatial and temporal conditions where tissue TF pairs play their regulation roles, tissue-type TF interactions provide a general view of their roles in multiple conditions. The analysis process has also led to other findings that the same type of tissues may have significant differences in both the contents of tissue TF pairs and the TF functional roles, which has been demonstrated by the conservation analysis of tissue TF pairs and their functions from muscle tissues. Tissue-type TF-TF interaction networks have provided not only lines of information on how tissue transcriptional programs are constructed but also new findings of potential roles for tissue-specific TFs in TF-TF interaction networks from liver tissue.

## Conclusions

In this study, we successfully employed our previously developed function conservation approach [22], to predict functional TF pairs from tissue-expressed genes in 79 human tissues. Based on the predictions, tissue TF pairs were identified. Our analyses led to the discovery of 2549 unique tissue TF pairs for the 79 human tissues. The validity of the discovered tissue TF pairs has been demonstrated by both known interacting and liver-specific TFs. We also extended our study to find interacting TFs controlling gene expression in a broad temporal and spatial manner and identified 379 tissue-type TF pairs from 11 tissue-type groups, from which tissue-type TF-TF interaction networks have been built. The results also indicated that tissue-specific TFs may play an important role in recruiting non-tissue-specific TFs to the TF-TF interaction network, offering the potential for coordinating and controlling tissue gene expression across a variety of conditions. In summary, our findings have shown that tissue gene expression is regulated by large sets of interacting TFs either on the same promoter of a gene or through TF-TF interaction networks.

## Methods

### Promoter sequences for housekeeping and tissue-expressed genes

The GNF Atlas2 gene expression database (gnfAtlas2) [34], which contains gene expression data from 79 human tissues, was used for the selection of genes. Based on the report of Pennacchio *et al *[18] we selected in each tissue the top 300 expressed genes (referred to tissue-expressed genes), which have been used and proven to successfully predict tissue-specific enhancers. Housekeeping genes are the 1018 genes defined by Farre *et al *[24]. Redundant genes in each group were first removed. Although regulatory elements can exist anywhere in the genome, they are more concentrated around the transcriptional start sites [35]. To reduce false predictions we focused on the proximal promoters which have been proven to successfully predict tissue-specific regulatory elements [21,36]. It is however worthy to note that the use of 1 kb promoter sequences has limitation for the prediction of tissue TF pairs when compared to the experimental approaches such as ChIP-chip experiment, in which TF pairs can be detected anywhere in the genome. Considering no benchmark promoter sequence dataset is currently available for computational prediction of functional TF pairs, the use of 1 kb promoter sequences and our computational approach nevertheless provide an alternative way for tissue interacting TF discovery. Promoter sequences within 1 kb upstream of transcriptional starting sites for both human and corresponding mouse orthologous genes were extracted from the UCSC genome browser (hg18 March 2006 assembly, mm9 July 2007 assembly). Orthologous genes with promoter sequences from both human and mouse were selected for further analysis. This procedure resulted in 208 to 278 orthologous promoter sequences for tissue-expressed genes and 986 orthologous promoter sequences for housekeeping genes.

### Prediction of TF pairs and tissue TF pairs

The procedures for predicting TF pair are basically the same as previously described [22] (Additional File 1). Briefly, background sequences were created by shuffling the DNA sequences within each promoter by either mixing completely or keeping dinucleotides together. These background sequences are preferable to using intergenic sequences which usually are AT-rich or exonic sequences whose nucleotide distributions tend to be biased, when compared to the test promoter sequences. The resulting shuffled sequences from human and mouse, together with the original promoter sequences, were employed for TFBS detection using the Match^® ^program [37], for which the profile parameter was set to "minimize the sum of false positives and negatives", and the 214 non-redundant vertebrate PWMs from the professional TRANSFAC11.4 database [25]. To detect enriched TF pairs out of 23,005 (214*215/2) possible combinations of 2 TFs, distance constraints were first applied for the selection of co-occurring TFBSs with a defined maximum distance between 2 TFBSs. A total of 10 distances were defined, ranging from the smallest 20 bp to the largest 200 bp with a 20 bp increment. The assumption behind the distance constraint is that functional TFBS pairs are more distance-restricted than random co-occurrence of TFBSs [19,38]. This is true not only in human, for which we found that functional TF pairs were enriched within 200 bp distance ranges [22], but also in Drosophila, in which short-range linkages (< 50 bp) between TFs was overrepresented but mid-range distances (100-500 bp) between TFs was depleted [39]. Enrichment of TFBS pairs for each distance constraint was achieved by computing the ratio of counts for a particular TFBS pair in real promoter sequences vs. the counts of the same TFBS pair in background sequences. To reduce noise while keeping as many as TFBS pairs for the integration of function conservation analysis described below, TFBS pairs with ratio > 1 in more than 5 distance constraints were selected.

A two-step analysis procedure was employed to compute the enrichment of overlapping orthologous genes for a particular TFBS pair. First, a cutoff threshold of at least 10% overlapping orthologous genes between mouse and human was set up for selecting genes whose promoters contained the TFBS pair. The enrichment of overlapping orthologous genes was then estimated by computing the ratio of overlapping orthologous genes from real promoter sequences against those from shuffled sequences. This analysis was performed for each distance constraint. The integration of function conservation for each TF pair was achieved by estimating the correlation (Pearson correlation coefficients) between the 10 enriched TFBS pairs and 10 corresponding enriched overlapping orthologous genes from the same distance constraint. Permutation tests were employed to estimate the statistical significance of correlation by randomly matching the 10 TFBS pair ratios with the 10 overlapping orthologous gene ratios. For multiple test correction, a cutoff threshold of *q*-value < 0.05 was applied. TF pairs are those passing the cutoff and common between human and mouse.

We next filtered TF pairs of housekeeping genes from those in each tissue (Figure 1a). This was done by removing TF pairs in a particular tissue common to those from housekeeping genes. The remaining TF pairs in each tissue were more tissue-specific, and therefore, were defined as tissue TF pairs. Similar results were obtained from using background sequences of either completely mixed nucleotides or keeping dinucleotides together or completely mixing nucleotides. The results from completely mixed nucleotides were used.

### Clustering analysis

To group tissues based on their tissue TF pairs, a 2549 (tissue TF pairs) × 79 (tissues) matrix with binary numbers was first built for all tissue TF pairs in the 79 human tissues. The presence of a tissue TF pair in the matrix was labeled with 1 and the absence was labeled with 0. A distance matrix was then built using the "binary" method, and hierarchical clustering was subsequently performed using the "complete" agglomeration method. All analysis was performed using the *R *statistical package [40].

### Predicting multiple interacting TFs

A two-step analysis of TFBS conservation and enrichment of overlapping orthologous genes was performed to predict interactions of 3 TFs. For TFBS conservation, the identified tissue TFBS pairs were first used to construct all possible 3-TFBS combinations by searching paired tissue TFBS pairs with one shared TFBS between each other on exactly the same location of a gene's promoter (Figure 1b) in a particular tissue. Orthologous gene pairs containing conserved 3-TFBS combination between human and mouse were then selected. Conserved 3-TFBS combinations are those whose 3 TFBSs have the same order and orientation on the promoter sequences between human and mouse orthologous genes. For enrichment of overlapping orthologous genes in a tissue, however, multiple interacting TFs from different orthologous gene pairs were considered to be the same as long as they contained the same 3 TFs. Enriched multiple interacting TFs are those with 3-TFBS combinations occurring on at least 10 orthologous gene promoters and with their target orthologous genes displaying significant overlap between human and mouse (*p *= 3 × 10^-2 ^to < 10^-36 ^and *q *< 0.05).

### Statistical methods for enrichment analyses

Two main statistical methods were employed for estimating the significance of enrichment in this study. For validating predicted TF pairs and tissue TF pairs by known interacting TFs, the binomial distribution probability, as shown below, was used to determine if known interacting TFs were present more often in the predicted TF pairs or tissue TF pairs than in a randomly selected group from a given list of TFs.

For example, in the case of estimating the statistical significance of known liver-specific interacting TFs in our predicted tissue TF pairs from liver tissue, the *n *is the number of known liver-specific interacting TFs in the predicted tissue TF pairs from this study; *N *the number of tissue TF pairs from liver tissue; and *p*_*f *_the background probability of liver-specific TF pairs in all possible combinations of 2 TFs from 214 PWMs.

The statistical significance was computed using the hypergeometric distribution to estimate (1) the enrichment of overlapping tissue TF pairs and their overlapping functions between muscle tissues, and (2) overlapping orthologous genes in predicting multiple interacting TFs.

In the case of overlapping orthologous genes in predicting multiple interacting TFs, for example, *c *is the number of orthologous gene pairs containing conserved 3-TFBS combination between human and mouse; *N *the number of tissue-expressed genes for a particular tissue; *S*_1 _and *S*_2 _are the numbers of tissue-expressed genes with 3-TFBS combinations corresponding to those in *c *for human and mouse, respectively. The resulting *p*-value is the probability of observing *c *or more orthologous gene pairs containing conserved 3-TFBS combination from two sets of size *S*_1 _and *S*_2 _drawn from a set of *N *tissue-expressed genes.

## List of abbreviations

TF: transcription factor; TFBS: transcription factor binding site; PWM: position weight matrices.

## Authors' contributions

ZH initiated and designed the study, conceived the analysis procedure, carried out data analysis, and wrote the manuscript. SG helped write Perl scripts to process data. Both authors read and approved the final manuscript.

## Supplementary Material

Additional file 1 Flowchart of analysis procedure for TF pair prediction. Click here for file

Additional file 2 Lists tissue and tissue-unique TF pairs and their potential functions for the 79 human tissues. Click here for file

Additional file 3 Lists tissue-type TF pairs for the 11 selected tissue groups. Click here for file

Additional file 4 Shows the 84 tissue-type TF-TF interaction networks from the 11 tissue-type groups. Click here for file

Additional file 5 Lists multiple interacting TFs (3 TFs) and their potential functions for the 79 human tissues. Click here for file

Additional file 6 **Overlap matrix for multiple interacting TFs.** The overlap of multiple interacting TFs between the 79 human tissues is depicted in the upper right panel and overlap of function for multiple interacting TFs in the lower left panel. The degree of overlap is indicated by color with red showing the greatest overlap and yellow showing less overlap. Click here for file
