# Supplementary figures and images for: Identification of interacting transcription factors regulating tissue gene expression in human
Source: BMC Genomics. 2010 Jan 19;11:49. doi: 10.1186/1471-2164-11-49 (PMC2822763; doi:10.1186/1471-2164-11-49)

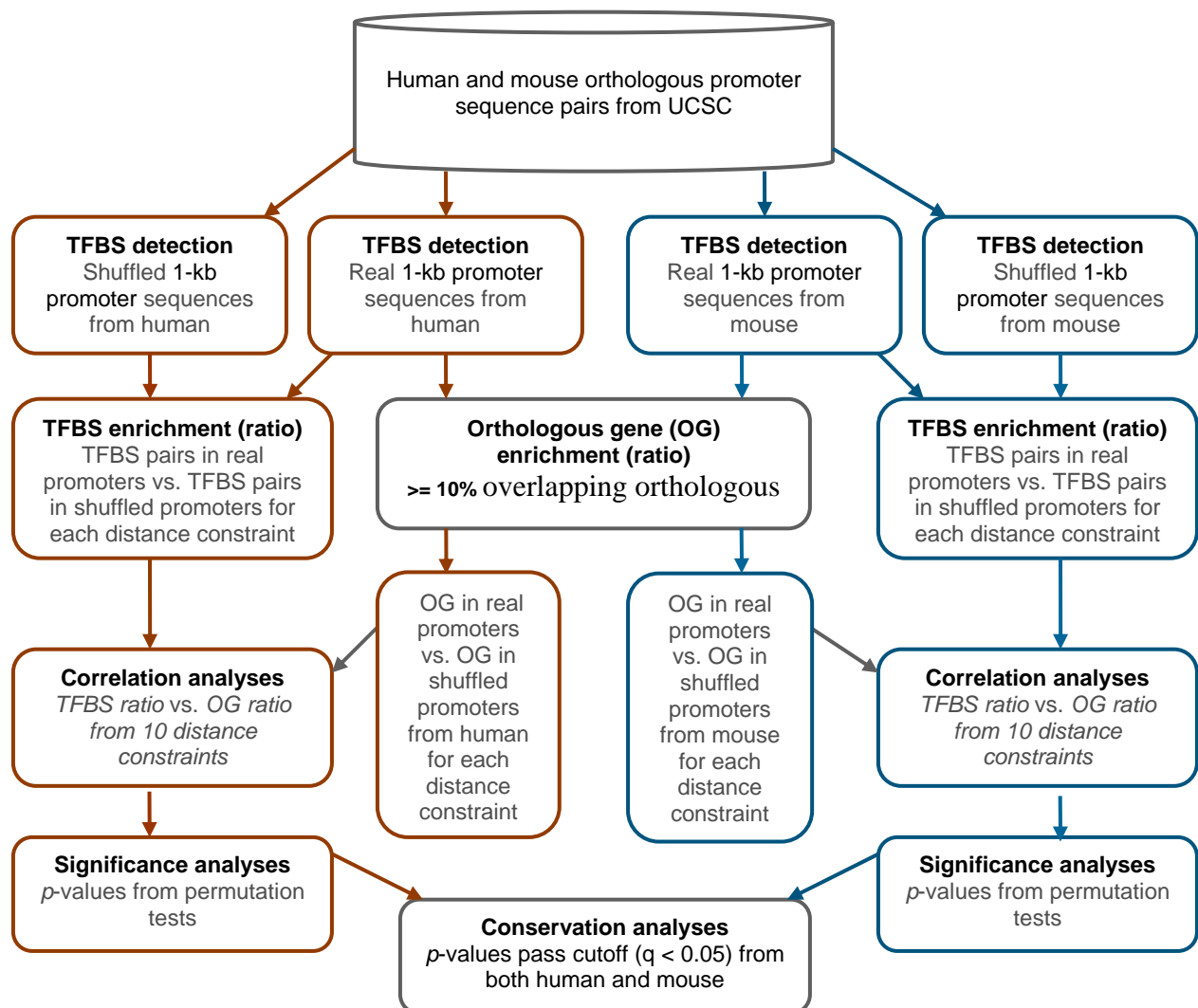

Flowchart of analysis procedure for TF pair prediction

Supplement: Additional file 1 — Flowchart of analysis procedure for TF pair prediction. [file 1471-2164-11-49-S1.pdf]

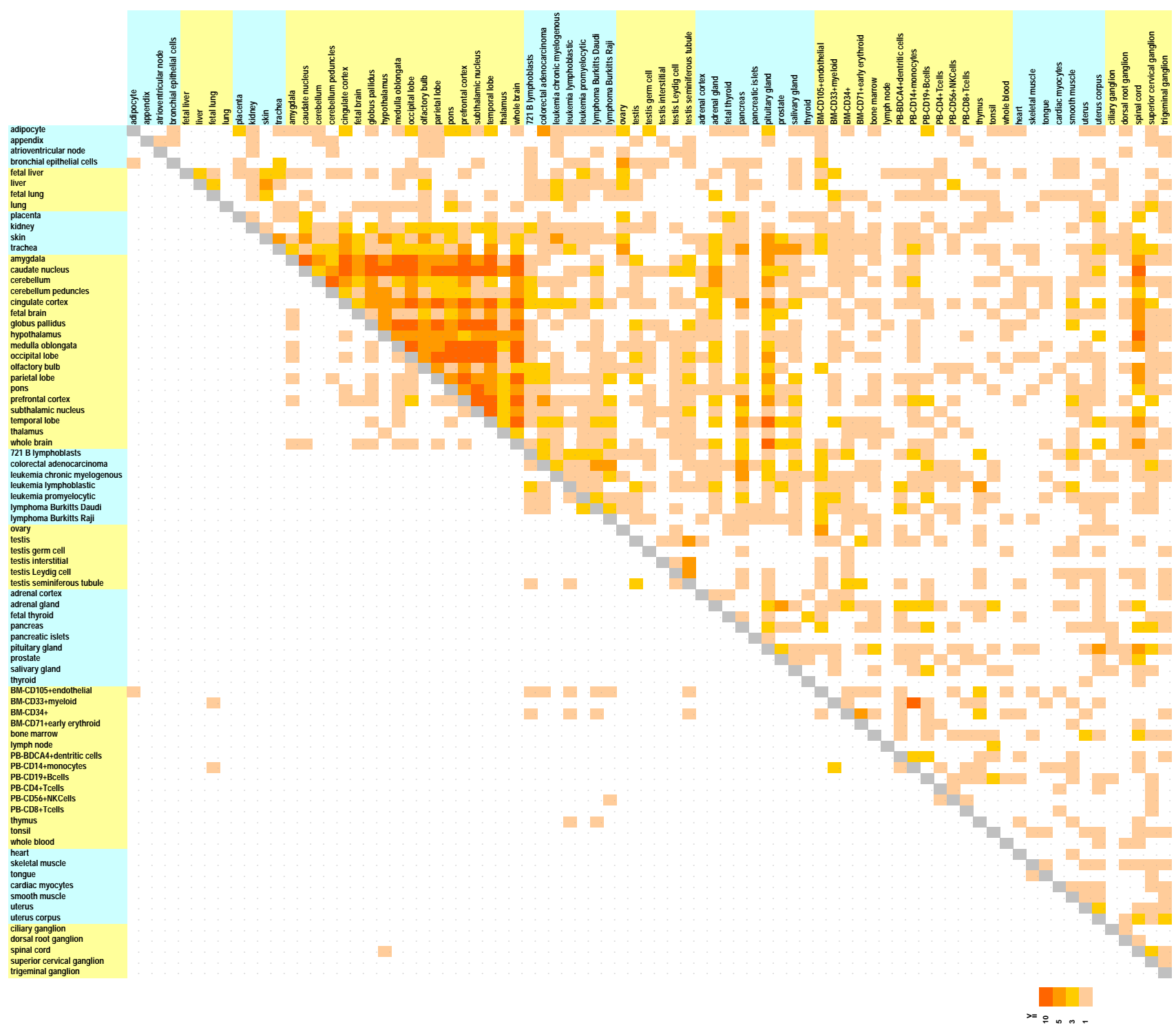

Supplement: Additional file 6 — Overlap matrix for multiple interacting TFs. The overlap of multiple interacting TFs between the 79 human tissues is depicted in the upper right panel and overlap of function for multiple interacting TFs in the lower left panel. The degree of overlap is indicated by color with red showing the greatest overlap and yellow showing less overlap. [file 1471-2164-11-49-S6.pdf]
