# Supplementary material for: Identification of interacting transcription factors regulating tissue gene expression in human
Source: BMC Genomics. 2010 Jan 19;11:49. doi: 10.1186/1471-2164-11-49 (PMC2822763; doi:10.1186/1471-2164-11-49)
Supplement: Additional file 4 — Shows the 84 tissue-type TF-TF interaction networks from the 11 tissue-type groups. [file 1471-2164-11-49-S4.pdf]

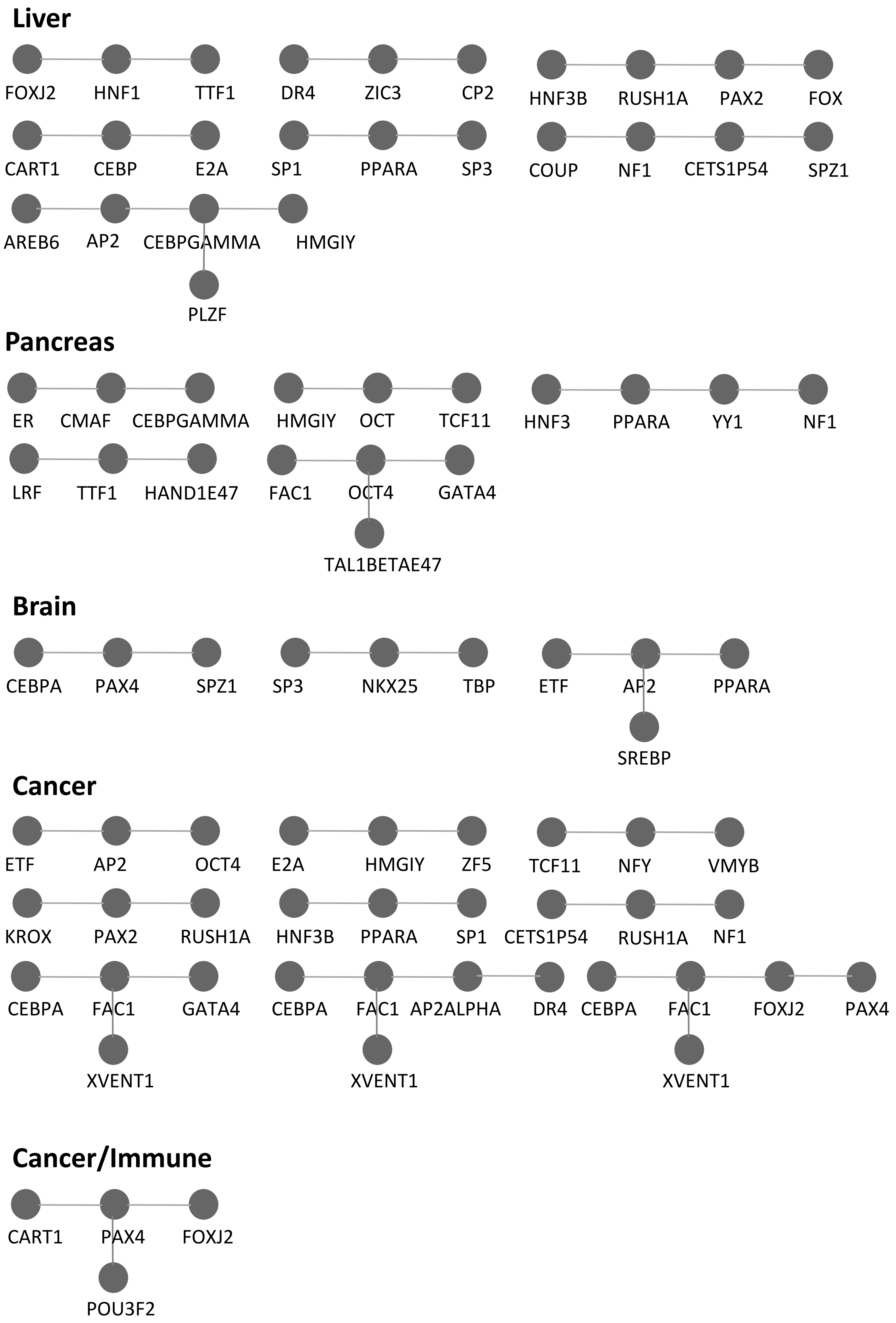

Liver
FOXJ2
HNF1
TTF1
DR4
ZIC3
CP2
HNF3B
RUSH1A
PAX2
FOX
CART1
CEBP
E2A
SP1
PPARA
SP3
COUP
NF1
CETS1P54
SPZ1
AREB6
AP2
CEBPGAMMA
HMGIY
PLZF
Pancreas
ER
CMAF
CEBPGAMMA
HMGIY
OCT
TCF11
HNF3
PPARA
YY1
NF1
LRF
TTF1
HAND1E47
FAC1
OCT4
GATA4
TAL1BETAE47
Brain
CEBPA
PAX4
SPZ1
SP3
NKX25
TBP
ETF
AP2
PPARA
SREBP
Cancer
ETF
AP2
OCT4
E2A
HMGIY
ZF5
TCF11
NFY
VMYB
KROX
PAX2
RUSH1A
HNF3B
PPARA
SP1
CETS1P54
RUSH1A
NF1
CEBPA
FAC1
GATA4
CEBPA
FAC1
AP2ALPHA
DR4
CEBPA
FAC1
FOXJ2
PAX4
XVENT1
XVENT1
XVENT1
Cancer/Immune
CART1
PAX4
FOXJ2
POU3F2

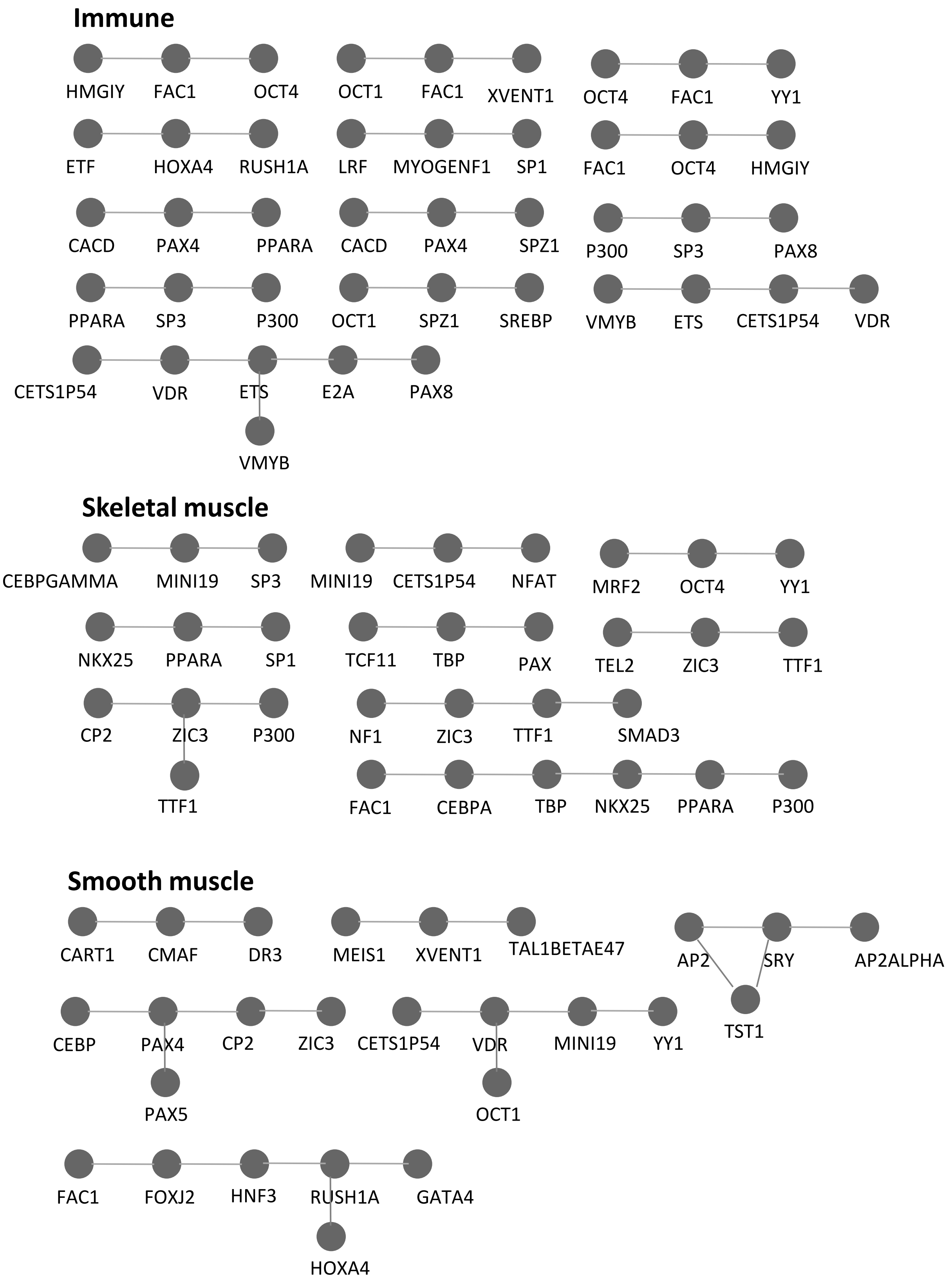
Immune
HMGIY FAC1 OCT4
OCT1 FAC1 XVENT1
OCT4 FAC1 YY1
ETF HOXA4 RUSH1A
LRF MYOGENF1 SP1
FAC1 OCT4 HMGIY
CACD PAX4 PPARA
CACD PAX4 SPZ1
P300 SP3 PAX8
PPARA SP3 P300
OCT1 SPZ1 SREBP
VMYB ETS CETS1P54 VDR
CETS1P54 VDR ETS E2A PAX8
VMYB
Skeletal muscle
CEBPGAMMA MINI19 SP3
MINI19 CETS1P54 NFAT
MRF2 OCT4 YY1
NKX25 PPARA SP1
TCF11 TBP PAX
TEL2 ZIC3 TTF1
CP2 ZIC3 P300
TTF1
NF1 ZIC3 TTF1 SMAD3
FAC1 CEBPA TBP NKX25 PPARA P300
Smooth muscle
CART1 CMAF DR3
MEIS1 XVENT1 TAL1BETAE47
AP2 SRY AP2ALPHA
TST1
CEBP PAX4 CP2 ZIC3
PAX5
CETS1P54 VDR MINI19 YY1
OCT1
FAC1 FOXJ2 HNF3 RUSH1A GATA4
HOXA4

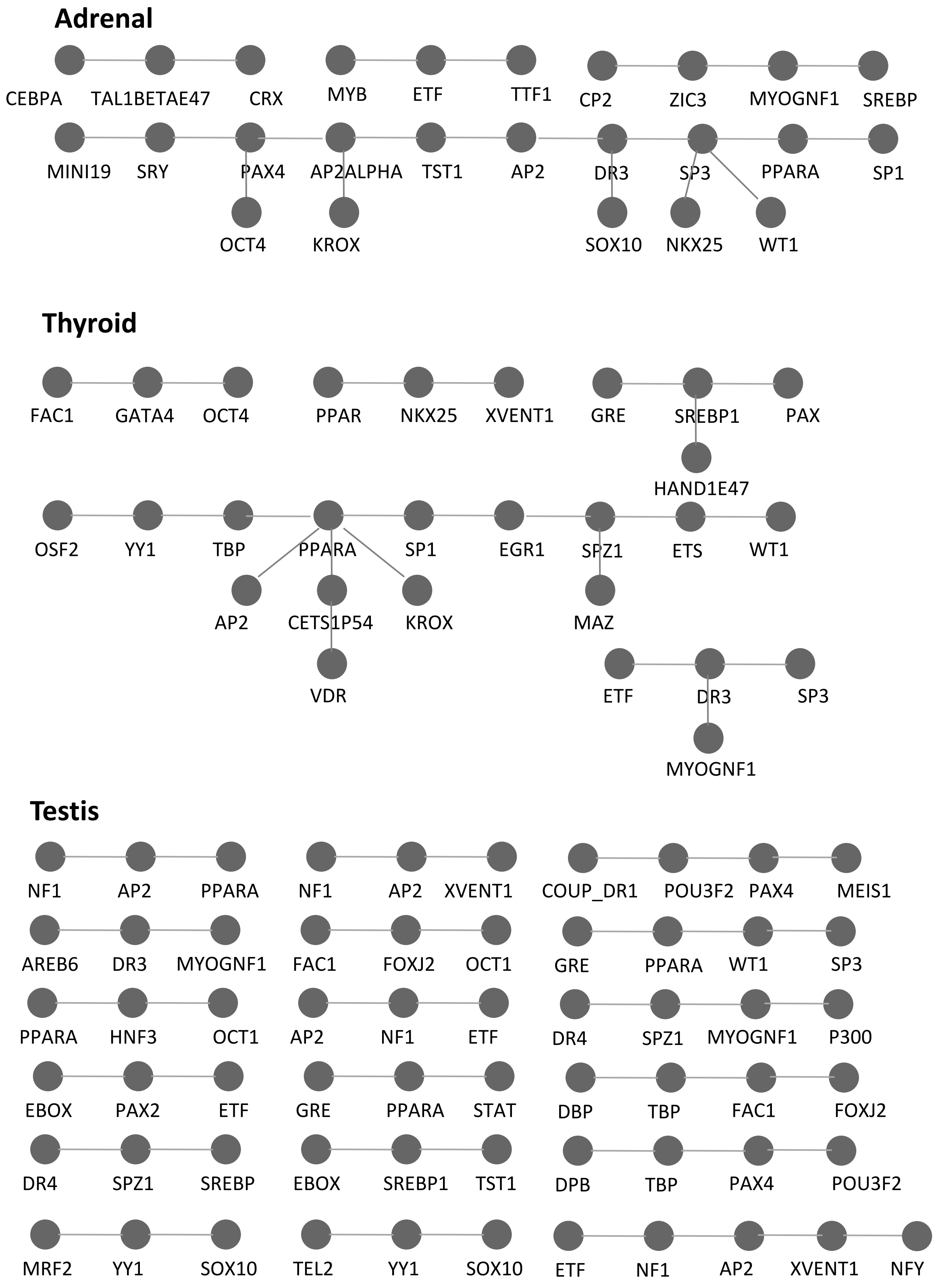

Adrenal
CEBPA
TAL1BETAE47
CRX
MYB
ETF
TTF1
CP2
ZIC3
MYOGNF1
SREBP
MINI19
SRY
PAX4
AP2ALPHA
TST1
AP2
DR3
SP3
PPARA
SP1
OCT4
KROX
SOX10
NKX25
WT1
Thyroid
FAC1
GATA4
OCT4
PPAR
NKX25
XVENT1
GRE
SREBP1
PAX
HAND1E47
OSF2
YY1
TBP
PPARA
SP1
EGR1
SPZ1
ETS
WT1
AP2
CETS1P54
KROX
MAZ
VDR
ETF
DR3
SP3
MYOGNF1
Testis
NF1
AP2
PPARA
NF1
AP2
XVENT1
COUP_DR1
POU3F2
PAX4
MEIS1
AREB6
DR3
MYOGNF1
FAC1
FOXJ2
OCT1
GRE
PPARA
WT1
SP3
PPARA
HNF3
OCT1
AP2
NF1
ETF
DR4
SPZ1
MYOGNF1
P300
EBOX
PAX2
ETF
GRE
PPARA
STAT
DBP
TBP
FAC1
FOXJ2
DR4
SPZ1
SREBP
EBOX
SREBP1
TST1
DPB
TBP
PAX4
POU3F2
MRF2
YY1
SOX10
TEL2
YY1
SOX10
ETF
NF1
AP2
XVENT1
NFY

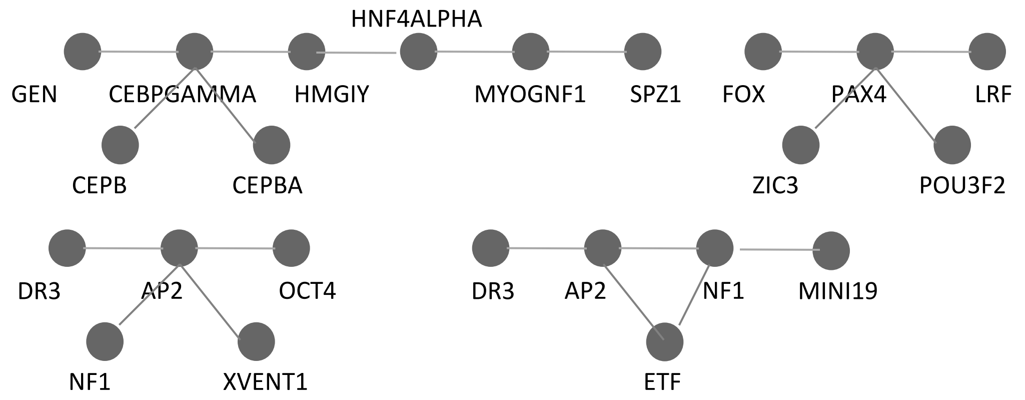

Additional data file 4. Tissue-type TF-TF interaction networks for the 11 selected groups. Out of 84 TF-TF interaction networks, 62 of them have a linear relationship between TFs, and the rest display more complex interacting structures with some of the TFs connecting to 3 to 5 TFs.
